# Femur neck fracture in young adults, is it really an urgent surgery indication: retrospective clinical study

**DOI:** 10.11604/pamj.2018.30.112.13643

**Published:** 2018-06-12

**Authors:** Seyitali Gumustas, Haci Bayram Tosun, Mehmet Isyar, Sancar Serbest, Kadir Oznam, Güven Bulut

**Affiliations:** 1 Dr Lutfi Kirdar Kartal Training and Research Hospital, Department of Orthopaedics and Traumatology, Istanbul, Turkey; 2 Department of Orthopaedics and Traumatology, Faculty of Medicine, Adiyaman University, Adiyaman, Turkey; 3 Medicalpark Bahcelievler, Department of Orthopaedics and Traumatology, Istanbul, Turkey; 4 Department of Orthopaedics and Traumatology, Faculty of Medicine, Kirikkale University, Kirikkale, Turkey; 5 Istanbul Medipol University School of Medicine, Department of Orthopaedic and traumatology, 34214, Istanbul, Turkey

**Keywords:** Timing of surgery, femoral neck fractures, internal determination, complication

## Abstract

**Introduction:**

Femur neck fracture comprises a significant part of intracapsular femur fracture in the intracapsular area of proximal femur and it is mostly seen in elder people. However, these kinds of fractures may be seen in young adults. The present study aims to search factors that affect femoral neck fractures in young adults after surgery carried out by internal determination method.

**Methods:**

Files of patients who were applied internal determination through closed reduction and cannulated screw because of intracapsular femur neck fractures between 2010 and 2015 were analyzed retrospectively. Fractures were evaluated by means of Garden classification, which is based on radiological appearance. The cases were examined in terms of timing of surgery in two groups. Cases operated in the first 24 hours after trauma consisted of group 1 and after 24 hours group 2. Radiological staging in femoral head avascular necrosis was evaluated by *Ficat-Arlet classification system* whereas acetabular fractures and hip functionality was evaluated by *Letournel and Judet system*, which is based on direct graph of fracture line.

**Results:**

Mean age at the time of surgery for 31 cases included in the study was 40.04 ± 9.63 year. The average duration from injury to surgery was 6.6 (1-20) days. Thirty nine percent of fractures was nondisplaced whereas 61% was displaced. The average follow-up period was 4.9 ± 1.35 years. The rate of nonunion was found 16% and femoral head avascular necrosis 6.4%. According to *Judet System,* 67.7% of cases showed excellent/good and 32.3% moderate/bad functional results. Six cases had a secondary surgery. Cases who had displaced fractures statistically showed worse functional results and underwent more secondary surgery than patients with nondisplaced fractures (P>0.05). As a result of logistic regression analysis, presence of displacement was a factor negatively affecting the judet score but did not affect the rate of complication. There were no significant differences between the two groups according to the surgical timing in terms of functional outcomes and complications.

**Conclusion:**

Because of surgical treatment of femoral neck fractures in the first 24 hours does not affect functional outcomes and complication rate, surgery is recommended in optimal conditions. In the case of displacement, care must be taken in terms of poor functional results.

## Introduction

Femoral neck fractures are treated surgically and accompanied by high mortality and morbidity, it is essential to use appropriate and efficient treatment approaches that comply with some parameters such as age, complexity of fractures and presence of other injuries [**1****-****3**]. Femur neck fractures substantially damage ascending cervical arteries of extracapsular arterial ring. The feeding of the femur head depends on the degree of damage that the retinal arteries have. Furthermore, all or a portion of the femoral head may become avascular [4]. Femoral neck fractures occur after a high-energy trauma in young adults [5]. Many researchers have reported two major complications following surgical treatment of femoral neck fractures; avascular necrosis of the femoral head (AVN) and bone nonunion [6-9]. The cases with AVN are affected in terms of their quality of life, moreover; treatment expenses constitute an extra economic burden on governments [10]. Many cases such as vascular damage, tamponade effect, fracture displacement, surgical treatment delay and surgical techniques have been investigated in these kinds of fractures [11]. The aims of surgical treatment in these fractures are anatomic reduction and stabilized fixation. When the literature is reviewed, important issues such as the timing of surgery and capsulotomy are not clear [12, 13]. In addition, it is reported that the success of a treatment depends on whether total anatomic reduction has been obtained, stability of fixation, type of fracture and bone quality [14-16]. The present study aimed to investigate the effects of age, timing of the surgery and presence of fracture displacement on complications and functional outcomes in young adults with femur neck fracture (FNF).

## Methods

Cases of one center were chosen retrospectively from 2010 to 2015. There were 47 cases whose ages ranged from 18-60. All patients were given detailed information about treatment and a written informed consent was obtained from each participant. A standard form was created where demographic data and clinical findings were recorded. Patients with pathologic fracture (n = 9) and fewer than 2 years of follow-up (n = 7) were excluded from the study. The cases were divided into two groups; those who were operated in the first 24 hours and after 24 hours after trauma. Fractures were evaluated using Garden Classification, which is based on radiological appearance [17]. According to this classification, cases with incomplete fracture line or impact fracture were classified as Type 1 and with complete but not displaced fracture as Type II. The cases with complete fracture and displacement rate was less than 50% were classified as Type III class and with complete fracture and displacement rate was more than 50% as Type IV. Insufficient fixation, loss of reduction or discernible fracture line for 12 months were described as nonunion [12]. Femoral head avascular necrosis was evaluated by Ficat criteria [18]. Functional results of hip was evaluated by Judet hip scores [19].

**Figure 1:**
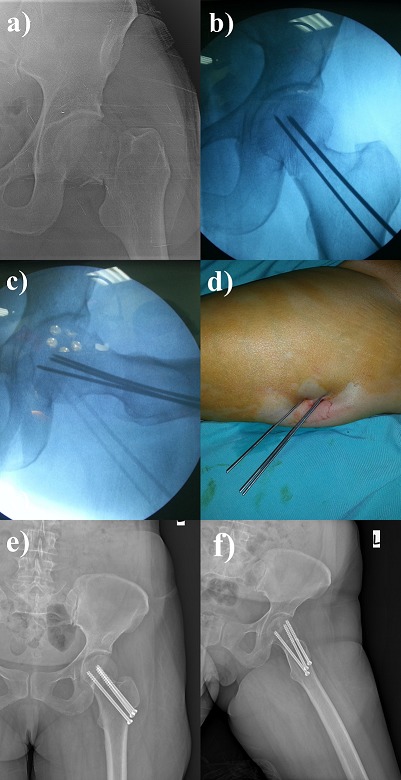
Radiographs of a 52 year-old woman with a left femoral neck fractures (Garden III fracture): Preoperative (a), intraoperative (b-c-d) and postoperative (e-f) radiographs

**Surgical technique**: The cases diagnosed with FNF were operated under general or spinal anesthesia on the traction table and applied internal fixation with 3 cannulated screws after closed reduction on fluoroscopic control as standard. Three half-grooved cannulated screws with 6.5 mm diameter were placed in longitudinal and equilateral triangle position (Figure 1). None of the cases were applied capsulotomy and/or joint aspiration. One hour before the operation, 1 mg cefazolin was started to all patients prophylactically and continued for 24 hours. Low-molecular-weight heparin started at post-op 12 hours and was applied for a total of 10 days. When operation notes were read it was understood that hip joint was allowed for active assisted movement the first day of post-op and the cases with no further problems were mobilized by allowing toe-touch with the help of walker and Canadian apparatus during the sixth week. It was found that mobilization was allowed after the 12th week but letting put impartial weight upon it.

**Statistical analyses**: The data were evaluated using the Statistical Package for the Social Sciences (version 20) program. Descriptive statistics are shown as mean-standard deviation or frequency (%). A 95% confidence interval was used to assess the data. The chi-square test was applied to categorical variables when comparing Judet functional assessment and rate of complication according to timing of surgery and the presence of displacement. The Mann Whitney u test was used while evaluating means for age according to the presence of complication and Judet classification. The factors affecting the Judet classification and the occurrence of complications were examined by logistic regression analysis. The alpha expressiveness value was regarded as <0.05.

## Results

Mean age of all cases was 39.51 ± 10.68 (min. 18 and max. 60 years). Forty two percent of whom (n = 13) were women and 45% (n = 14) of the factures were on the right side. According to Garden classification, the percentages of the types were as follow: 13% (n = 4) Type-1, 26% (n = 8) Type-2, 19% (n = 6) Type-3 and 42% (n = 13) Type 4. Thirty nine percent of fractures (n = 12) were nondisplaced (Garden Tip 1-2) and 61% (n = 19) displaced (Garden Tip 3-4). Additional fracture was present in 10 patients. There was an additional fracture (1 upper, 3 lower extremities and 3 pelvis fractures) in 7 patients and 3 patients had multitrauma. Five patients had controlled diabetes, 7 patients had controlled hypertension. The average time to surgery after trauma was 6.6 days (min. 1 and max. 20 days). Eighty four percent of the cases (n = 26) had a total bone union and time for bone union ranged from 4 and 11 months with the average of 6.01 months. The rate of nonunion was found 16% (n = 5) and femoral head avascular necrosis 6.4% (n = 2). No wound infection occurred in any of the patients. There were no significant differences between the groups with and without complications in terms of mean age and timing of surgery (p>0.05). The rate of complication or need for secondary surgery in displaced fractures was significantly higher than that of nondisplaced (p = 0.03) (Table 1). Logistic regression analysis showed no significant effect of displacement on the complication rate (B = 20.430; p = 0.999). Six cases were required a secondary surgery. In two year time (6m-4y), 4 cases due to nonunion, 1 case due to femur head AVN and 1 case due to nonunion and femur head AVN were applied total hip arthroplasty. According to Judet functional scores [19]; 18 cases (57%) wonderful, 3 cases (10%) good, 4 case (13%) moderate and 6 cases (20%) had bad functional results. When comparing, Judet functional results were taken in two groups. Timing of the surgery and means for age did not show difference between the two group. The rate of bad functional results in cases with displaced fractures was significantly higher than cases with nondisplaced (p=0.046) (Table 2). According to logistic regression analysis, presence of displacement was a factor that negatively affected Judet functional score (B = 2.293; p = 0.045).

**Table 1:** Rate of complication/secondary surgical intervention according to patient’s age, timing of the surgery, and presence of displaced fracture

			Presence of Complication / Secondary İntervention		p
			-	+	
Age (Year)		Mean (±SD)	38,64 (10,86)	43,16 (9,90)	0,36
Timing of Surgery	≤ 24 h	n (%)	11 (78,6)	3 (21,4)	0,79
	>24 h	n (%)	14 (82,4)	3 (17,6)	
Presence of Displacement	Non-displaced	n (%)	12 (100)	0 (0)	0,03*
	Displaced	n (%)	13 (68,4)	6 (31,6)	

* p˂0,05

**Table 2:** Judet functional result scores according to patient’s age, timing of the surgery, and presence of displaced fracture

			Functional Scores		*p*
			Excellent / Good	Moderate / Poor	
Age (Year)		Mean (±SD)	37,6(11,3)	43,4(8,4)	0,128
Timing of Surgery	≤ 24 h	n(%)	10(71,4)	4(28,6)	0,69
	>24 h	n(%)	11(64,7)	6(35,3)	
Presence of Displacement	Non-displaced	n(%)	11(91,7)	1(8,3)	0,046*
	Displaced	n(%)	10(52,6)	9(47,4)	

* p˂0,05

## Discussion

We aimed to determine whether factors such as age, timing of surgery and presence of displacement affected the rate of complications and Judet functional scores in patients with femur neck fractures. It is reported in literature that the aim of treatment in young adults with femoral neck fractures is to provide fracture healing, inhibiting osteonecrosis by preserving femur head and thus rendering a fast rehabilitation for the purpose of enabling patient to restoring his/her health [20, 21]. There is a study in which the importance of early surgical treatment, anatomic reduction and stable fixation [22]. Furthermore, in researches it is mentioned that the stability of the fracture and individual differences in selecting treatment modalities play an important role [4,20, 23]. Treatment of femoral neck fractures is closely related to high morbidity at almost any age, so it is underlined that more attention is required in the treatment of young adults due to complications such as femoral head necrosis and nonunion [24]. It was reported that nonunion rates were 10%-30% after femur neck fracture, yet femoral head necrosis rates were 15%-30% [4, 23, 25]. When literature is reviewed, it is maintained that the relation between nonunion and femoral head necrosis was not fully understood [26]. In literature, there are some studies indicating when an early intervention is required, through which methods a full anatomic reduction and stable fixation could be obtained and whether capsulotomy or joint aspiration in order to diminish pressure in the capsule, yet they have not achieved a consensus [4, 22, 27-30]. Also, cultural characteristics, race, nutrition and age are some of the predictive factors in femoral neck fractures [27]. In the present study we observed that nonunion rate was 16%, which shows resemblance to the studies in literature. However, on the contrary to these studies [4, 22, 27-30], we observed that femoral head necrosis rate was much lower (6.4%). Schweitzer et al. reported that femoral head necrosis rates in patients range from 50 to 65 years old are more when compared to younger patients, and this is not related to timing of the surgery. It is indicated that age is a predictive factor for femoral head necrosis [27]. However, we could not find any significant difference means for age between the patients with complications such as nonunion and femoral head necrosis and those without complications. It is underlined that early diagnosis is crucial for the purpose of restoring femoral head support in intracapsular femur fractures in young adults [26, 31]. Moreover, it is stated that early intervention is an essential factor in reaching ideal results [22, 32, 33]. On the other hand, femur neck fractures mostly occur due to high energy trauma and accompanied with various multisystem injuries in young adults. Because of time span for other system problems, it is not always possible to have an early surgery in femur neck fractures [20, 22]. The situation about surgical timing is controversy and some data are lack of proof [28, 29]. Some studies suggest that early surgery within 6-24 hours might decrease femur head femoral head necrosis [34, 35]. Avascular necrosis rates are reported to have different values [22, 34-36].

Although some studies indicating that avascular necrosis rate in cases under 60 years old and who are undergone early fixation during the first 12 hours [34], on the other hand, others indicate that internal fixation should be applied in the first 24 hours in order to diminish risk of complication [22, 36]. Braun et al state that fixation applied during the first 6 hours improves both functions and avascular necrosis rates [37], yet in some other researches it is reported that there is no relation between nonunion or avascular necrosis development risk and timing of the surgery [23, 38, 39]. Razik et al [28] report that there is no evidence supporting the general belief that early surgical fixation decreases AVN risk. Upadhyay et al [23] maintain that fixation during and after first 48 hours does not affect bone nonunion and femur head avascular necrosis rate whereas Haidukewych et al [38] accept it during and after first 24 hours. Elmi et al [39]; claim that timing of the surgery has an important role yet it is not only the factor as bone quality of patients, reduction status and metabolic and nutritional parameters are essential as well. We could not find a significant difference in the complication rates according to the surgical timing similar to the literature. Researchers infer that there is a strong bond between femur head avascular necrosis and displacement of fracture [39, 40]; the rate of avascular necrosis is lower in nondisplaced fractures than displaced fractures [41]. We have found a significantly higher complication rate in displaced fractures compared to nondisplaced fractures, similar to the literature. But as a result of logistic regression analysis, we could not find the presence of displacement as a factor predicting the complication. It is mentioned that capsulotomy application is a controversy issue in femur neck fractures and it varies from one situation to another [22]. There are some views supporting that capsulotomy or aspiration decreases intraarticular pressure following femur neck fractures, so it increases blood stream for each femur and decreases ischemia risk by inducing an increase in the perfusion [42]. Parker et al report that hematoma aspiration can be applied during internal fixation, yet there isn't any proof to justify capsulotomy [4]. Ly et al. suggest that this process causes time waste and risk and what's more it causes femur head avascular necrosis in a small part of patients [22]. It is found that secondary surgery rate was between 33% - 52% after internal fixation of displaced intracapsular femur neck fractures [23, 38, 43]. In our research, there hasn't been any evidence that cases were applied capsulotomy or hematoma aspiration. In line with the literature 31.5% of the displaced fractures were applied hip arthroplasty as secondary surgery. In our study, evaluation of functional results were performed with Judet's scoring system, because it is a simple and comprehensive scoring system. According to timing of surgery, results did not show differences and there was no significant difference in mean age between the two groups with good and poor Judet scores. Presence of displacement was found to be a predictor of poor functional outcome as a result of logistic regression. A study demonstrate that even delayed fixation of displaced femoral neck fractures in young adults is associated with a high rate of fracture union [44]. The findings of this study regarding the timing of surgery are similar to our study, whereas our findings regarding the relationship between displacement and functional outcomes differ. Being a retrospective research and having small number of cases are the most important limitations of our study.

## Conclusion

A surgical protocol with anatomical reduction and internal fixation applied under ideal conditions has a vital importance in the treatment of femoral neck fractures. It is recommended to be careful in terms of complications and functional outcomes in cases with displaced fractures. Surgical intervention is recommended when optimum conditions are provided instead of emergent surgery after trauma.

### What is known about this topic

- The aims of surgical treatment in these fractures are anatomic reduction and stable fixation. When the literature is reviewed, timing and capsulotomy in the surgical treatment are not clear;
- It is stated that early intervention is an essential factor in reaching ideal results;
- There are some views supporting that capsulotomy or aspiration decreases intraarticular pressure following femur neck fractures, so it increases blood stream for each femur and decreases ischemia risk by inducing an increase in the perfusion. But, it is mentioned that capsulotomy application is a controversy issue in femur neck fractures.

### What this study adds

- In this study, we could not find a significant difference between complication rates and the surgical timing;
- Hematoma aspiration and capsulotomy don't affect the results of the femoral neck fractures;
- There is a significantly higher complication rate in displaced fractures compared to nondisplaced fractures. Surgical intervention is recommended when optimum conditions are provided instead of emergent surgery after trauma. There is no evidence supporting the general belief that early surgical fixation decreases AVN risk.
